# Sarcopenic Obesity in Cervical Carcinoma: A Strong and Independent Prognostic Factor beyond the Conventional Predictors (ESTHER Study—AFRAID Project)

**DOI:** 10.3390/cancers16050929

**Published:** 2024-02-25

**Authors:** Federica Medici, Martina Ferioli, Silvia Cammelli, Ludovica Forlani, Viola Laghi, Johnny Ma, Savino Cilla, Milly Buwenge, Gabriella Macchia, Francesco Deodato, Maria Vadalà, Claudio Malizia, Luca Tagliaferri, Anna Myriam Perrone, Pierandrea De Iaco, Lidia Strigari, Alberto Bazzocchi, Stefania Rizzo, Alessandra Arcelli, Alessio Giuseppe Morganti

**Affiliations:** 1 Department of Medical and Surgical Sciences (DIMEC), Alma Mater Studiorum University of Bologna, 40138 Bologna, Italy; martina.ferioli4@unibo.it (M.F.); silvia.cammelli2@unibo.it (S.C.); ludovica.forlani@studio.unibo.it (L.F.); viola.laghi@studio.unibo.it (V.L.); johnny.ma@studio.unibo.it (J.M.); mbuwenge@gmail.com (M.B.); myriam.perrone@aosp.bo.it (A.M.P.); pierandrea.deiaco@unibo.it (P.D.I.); alessandra.arcelli@aosp.bo.it (A.A.); alessio.morganti2@unibo.it (A.G.M.); 2 Radiation Oncology, IRCCS Azienda Ospedaliero—Universitaria di Bologna, 40138 Bologna, Italy; 3 Medical Physics Unit, Gemelli Molise Hospital—Università Cattolica del Sacro Cuore, 86100 Campobasso, Italy; savinocilla@gmail.com; 4 Radiotherapy Unit, Gemelli Molise Hospital, Fondazione Policlinico Universitario A. Gemelli, IRCCS, 86100 Campobasso, Italy; gabriella.macchia@gemellimolise.it (G.M.); francesco.deodato@unicatt.it (F.D.); 5 Nuclear Medicine, IRCCS Azienda Ospedaliero—Universitaria di Bologna, 40138 Bologna, Italy; maria.vadala@gmail.com (M.V.); claudio.malizia@aosp.bo.it (C.M.); 6 UOC di Radioterapia Oncologica, Dipartimento Diagnostica per Immagini, Radioterapia Oncologica ed Ematologia, Fondazione Policlinico Universitario Agostino Gemelli IRCCS, 00168 Roma, Italy; luca.tagliaferri@policlinicogemelli.it; 7 Division of Gynecologic Oncology, IRCCS Azienda Ospedaliero—Universitaria di Bologna, 40138 Bologna, Italy; 8 Medical Physics, IRCCS Azienda Ospedaliero—Universitaria di Bologna, 40138 Bologna, Italy; lidia.strigari@aosp.bo.it; 9 Diagnostic and Interventional Radiology, IRCCS Istituto Ortopedico Rizzoli, 40136 Bologna, Italy; alberto.bazzocchi@unibo.it; 10 Service of Radiology, Imaging Institute of Southern Switzerland, Ente Ospedaliero Cantonale (EOC), CH-6500 Lugano, Switzerland; stefania.rizzo@eoc.ch

**Keywords:** anemia, body mass index, chemoradiation, cervical cancer, hemoglobin, observational study, overall survival, predictive model, sarcopenia, sarcopenic obesity

## Abstract

**Simple Summary:**

This study investigates how the assessment of body mass index (BMI), together with body fat and muscle mass, might influence the success of treatments for advanced cervical cancer. Unlike most research which focuses on general health and cancer stages, this research pays particular attention to the patient’s weight-to-height ratio, muscle mass, and especially the condition known as sarcopenic obesity—a combination of high body fat and low muscle mass. The findings reveal that sarcopenic obesity significantly affects patient outcomes, suggesting that it could be a key factor in deciding the best course of treatment. Recognizing these body composition indicators could lead to more personalized and effective treatment plans, marking a step forward in the way we approach care for cervical cancer patients.

**Abstract:**

Locally advanced cervical cancer represents a significant treatment challenge. Body composition parameters such as body mass index, sarcopenia, and sarcopenic obesity, defined by sarcopenia and BMI ≥ 30 kg/m^2^, have been identified as potential prognostic factors, yet their overall impact remains underexplored. This study assessed the relationship between these anthropometric parameters alongside clinical prognostic factors on the prognosis of 173 cervical cancer patients. Survival outcomes in terms of local control (LC), distant metastasis-free survival (DMFS), disease-free survival (DFS), and overall survival (OS) were analyzed using Kaplan regression methods—Meier and Cox. Older age, lower hemoglobin levels, higher FIGO (International Federation of Gynecology and Obstetrics) stages, and lower total radiation doses were significantly associated with worse outcomes. Univariate analysis showed a significant correlation between BMI and the outcomes examined, revealing that normal-weight patients show higher survival rates, which was not confirmed by the multivariate analysis. Sarcopenia was not correlated with any of the outcomes considered, while sarcopenic obesity was identified as an independent negative predictor of DFS (HR: 5.289, 95% CI: 1.298–21.546, *p* = 0.020) and OS (HR: 2.645, 95% CI: 1.275–5.488, *p* = 0.009). This study highlights the potential of sarcopenic obesity as an independent predictor of clinical outcomes. These results support their inclusion in prognostic assessments and treatment planning for patients with advanced cervical cancer.

## 1. Introduction

Cervical cancer is a widespread global malignancy [1]. In the case of locally advanced cervical cancer (LACC), the standard treatment is concurrent chemoradiation (CRT), involving the simultaneous use of chemotherapy (CHT) and radiotherapy (RT) [1]. While CRT effectively controls the local tumor in many cases, about one third of patients show treatment failure during the follow-up [2,3].

In recent years, there has been a growing interest in developing predictive models for cancer outcomes [4]. These models have the potential to help clinicians anticipate how patients will respond to specific treatments, allowing for more personalized medical interventions tailored to individual factors like cancer stage, recurrence risk, and patient demographics.

Several factors have been identified in the context of LACC as predictors of overall survival (OS) [5,6]. These factors include tumor size, histological type, lymph node metastases, and the Federation of Gynecology and Obstetrics (FIGO) stage. Anemia has also been recognized as a negative prognostic factor for LACC patients [7,8,9,10].

To improve the accuracy of outcome predictions and enable treatment customization based on prognostic profiles, recent studies have explored the use of various body composition-related parameters in cancer patients. In particular, there is a growing awareness of the impact of parameters such as body mass index (BMI), sarcopenia (SP), and sarcopenic obesity (SO) in cancer patients undergoing RT [11,12].

Also, in LACC, several studies have explored the impact of SP in patients treated with CRT. Kiyotoki et al. found that SP, defined as a ≥15.0% loss of iliopsoas muscle from baseline, was a significant independent prognostic factor for disease-free survival (DFS) and OS in CRT patients [13]. Similarly, Abe et al. emphasized the importance of maintaining muscle mass and quality during treatment, as extreme leanness, lower skeletal muscle quality, and muscle loss during therapy predicted poor prognosis [14]. Moreover, Lee et al. identified skeletal muscle loss as an imaging biomarker associated with outcomes after definitive CRT for LACC without specifically addressing SP [15].

Moving on to the relationship between BMI and LACC outcomes, Gnade et al. observed that obese and morbidly obese women had a disproportionately inappropriate screening before cervical cancer diagnosis, and morbidly obese women exhibited worse OS [16]. Moreover, Clark et al. found that both extremes of BMI (underweight and overweight/obesity) were associated with worse OS in LACC [17]. Additionally, Münstedt et al. did not find a negative impact of obesity on the prognosis of patients with LACC but observed that a higher BMI was associated with improved OS for endometrial and cervical carcinomas [18].

Finally, studies have explored the significance of the prognostic nutritional index (PNI = (10 × serum albumin [g/dL]) + (0.005 × lymphocytes/μL)) in LACC patients. Haraga et al. found that low PNI predicted poor prognosis in LACC patients undergoing CRT and RT [19]. In another study, Wang et al. supported the importance of PNI and systemic inflammatory indexes as predictors of prognosis in patients with stage IIB-III LACC receiving RT [20]. Furthermore, Guo et al. demonstrated that a low PNI was associated with lower quality of life, reduced tolerance to RT and CHT, lower objective response rates, and worse OS for LACC [21].

However, many of these studies have mainly focused on just one anthropometric parameter [16,17,18] or a limited set of such indices [13,19,20,21]. Furthermore, the impact of SO in LACC has not been previously evaluated and several studies did not thoroughly examine potential confounding variables [18,19]. Therefore, the primary aim of this study is to investigate the predictive capabilities of a range of body composition-related parameters in a large group of LACC patients. This investigation considered relevant clinical prognostic factors, including clinical information, tumor-related characteristics, and treatment-related data. This analysis was conducted by a multidisciplinary team comprising radiation oncologists, radiologists specializing in body composition, medical physicists, RT technicians, and gynecological oncologists.

## 2. Materials and Methods

### 2.1. Study Objective and Design

The primary objective of this investigation was to assess the relationship between various nutrition-related parameters and the prognosis of LACC. This was achieved by examining their impact on key clinical outcomes, including local control (LC), distant metastasis-free survival (DMFS), DFS, and OS. To accomplish this, we conducted a retrospective analysis of patients who received treatment at our institution from July 2007 to July 2021. These patients were part of an approved observational study known as the ESTHER study, which was conducted in accordance with the guidelines set by our local Ethical Committee (code CE 973/2020/Oss/AOUBo). Moreover, this analysis is part of our institutional AFRAID (impAct oF saRcopeniA In raDiotherapy) project [11]. All patients provided informed consent to participate in this study, and no specific exclusions were applied to ensure the real-world relevance of the research.

### 2.2. Staging, Treatment, and Follow-Up

LACC staging followed the 2018 FIGO system. The FIGO stage was evaluated using a comprehensive approach that included whole-body CT with contrast medium, pelvic MRI, and examination under anesthesia. Additionally, the FIGO stage was confirmed by ^18F^-Fluorodeoxyglucose Positron Emission Tomography/Computed Tomography performed for radiotherapy planning purposes. Patients received definitive CRT with pelvic external beam RT (45–50 Gy in 1.8–2 Gy fractions) and intracavitary BRT (80–90 Gy for the gross tumor volume). The clinical target volume included relevant areas with specific expansions, as previously described [22,23]. Patient alignment was monitored daily with imaging devices [24]. Chemotherapy involved weekly intravenous Cisplatin (40 mg/m^2^). Follow-up included physical exams every three months for the first two years, followed by six-month intervals for three years. Computed tomography scans were performed as needed or every six months in the first two years and annually thereafter [22,23].

### 2.3. Examined Parameters

#### 2.3.1. Patient, Tumor, and Treatment Information

In this analysis, we included patient-related factors such as age and hemoglobin (Hb) levels (measured in g/100 mL). We also considered tumor-related details, such as histological type, FIGO stage, clinical tumor stage, clinical nodal stage, and maximum tumor diameter. Furthermore, we incorporated treatment-related data, including RT technique, external beam RT dose and fractionation for the pelvic region, BRT boost dose, total tumor dose, and overall treatment time measured in days.

#### 2.3.2. Body Composition Parameters

**Figure 1:**
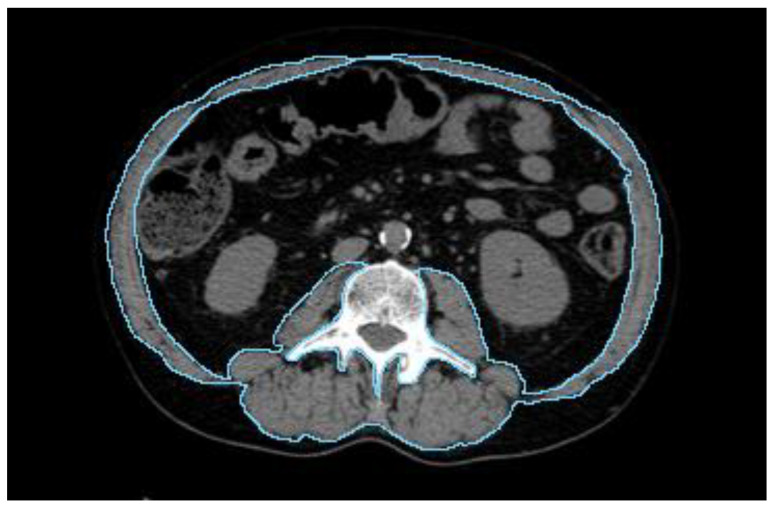
Delineation of the skeletal muscle area on a CT scan at the level of the third lumbar vertebra. Adapted from Medici F et al., 2022 [11].

The analysis encompassed the evaluation of various body composition-related parameters, including BMI, SP, and SO. BMI was calculated as the ratio of an individual weight in kilograms to the square of their height in meters (BMI = weight in kg/(height in m)^2^) and it was stratified into four categories (underweight—BMI <18.5 kg/m^2^; normal weight—18.5 to 24.9 kg/m^2^; overweight—25 < BMI < 29.9 kg/m^2^; and obesity—BMI ≥ 30 kg/m^2^) according to the cut-off points proposed by Weir et al. [25]. SP was calculated as follows. We collected all pre-treatment CT scans to perform an instrumental evaluation of the skeletal muscle area (SMA). ‘At the level of the third lumbar vertebra (L3), we identified and contoured all relevant muscle structures, including the rectus abdominis, external oblique, internal oblique, transversus abdominis, quadratus lumborum, spinalis, longissimus thoracis, iliocostalis lumborum, and psoas muscles. This comprehensive assessment is based on the established correlation between muscle mass at this level and overall body muscle volume, as commonly performed in the field’ [26], demonstrating the method reliability for evaluating SP [27] (Figure 1). The defined region of interest (SMA) was then divided by body surface area, obtaining the skeletal muscle index (SMI) [28]. SP patients were defined by using a cut-off established by calculating the median skeletal muscle index (SMI) value minus 2 standard deviations. Moreover, we derived the cut-off from Prado and Martin to establish if the sarcopenic condition was present or not [28,29]. Finally, SO was defined by assessing the prevalence of SP alongside the co-presence of a BMI ≥ 30 kg/m^2^, aligning with the criteria set forth by Prado et al. [28] in their landmark study on the prevalence and clinical implications of sarcopenic obesity in patients with solid tumors. 

### 2.4. Statistical Analysis

We used basic statistics to describe patient and tumor characteristics, along with treatment details. Categorical data are shown as numbers and percentages, while continuous data are presented as median values and their ranges. We calculated several time-related outcomes: (I) LC: the time from the start of concurrent CRT until local-regional recurrence was detected or the last follow-up for patients without pelvic recurrence. (II) DMFS: the period from the initiation of CRT until distant failure was identified or the last follow-up for patients without distant recurrence. (III) DFS: the time from CRT initiation until any treatment failure occurred or until the last follow-up for patients without LACC recurrence. (IV) OS: the interval between CRT initiation and either the time of death or the most recent follow-up date. We analyzed these outcomes using survival curves generated by the Kaplan–Meier method and performed a simple comparison (log-rank analysis) that included all the mentioned variables. Additionally, we conducted a multivariate Cox’s regression analysis, including variables with a *p*-value of less than 0.1 from the univariate analysis. We considered a significance level of 5% (*p* < 0.05). Our analysis was carried out using SPSS for Windows (version 20.0; SPSS Inc., Chicago, IL, USA).

## 3. Results

### 3.1. Patient Demographics

In this analysis, a total of 173 patients were involved. Table 1 provides comprehensive details about the patients. The median age at diagnosis was 56 years, ranging from 27 to 85 years, and the median follow-up duration was 36 months, spanning from 3 to 151 months.

### 3.2. Treatment Details

Treatment details are shown in Table 1. All patients underwent concurrent CRT with weekly Cisplatin administration. For patients with positive lymph nodes (57 cases), an additional dose was given either sequentially or simultaneously, resulting in a median total dose of 57.5 Gy (ranging from 52.5 to 61.0 Gy). All patients received BRT, with a median dose of 37 Gy for Pulsed-Dose-Rate BRT (ranging from 23 to 39 Gy) and 28 Gy for High-Dose-Rate BRT (ranging from 4 to 42 Gy).

### 3.3. Univariate Analysis

Regarding patient-related factors, advanced age was significantly associated with poorer DMFS (*p* = 0.049) and OS (*p* = 0.003). Lower pretreatment Hb levels were significantly linked to worse LC (*p* < 0.001), DFS (*p* = 0.007), and OS (*p* = 0.040). In terms of tumor-related parameters, our analysis revealed the following significant correlations: patients with lymph node metastases had worse DMFS (*p* = 0.045), and subjects with more advanced FIGO stages exhibited worse LC (*p* = 0.005), DMFS (*p* = 0.021), DFS (*p* = 0.003), and OS (*p* = 0.032). However, no significant differences were observed regarding maximum tumor diameter and histological type. Regarding treatment factors, higher total RT doses were significantly correlated with improved OS (*p* = 0.012), while no significant differences were noted based on treatment duration. The stratification of patients based on SP did not reveal any statistically significant correlations with the considered outcomes. Moreover, BMI was significantly correlated with DMFS (*p* = 0.033), DFS (*p* = 0.018), and OS (*p* = 0.023), with the highest rates recorded in normal-weight patients. Finally, patients with SO had a significantly worse DFS (*p* = 0.005) and OS (*p* < 0.001) in comparison with patients without this condition (Table 1).

### 3.4. Multivariate Analysis

**Figure 2:**
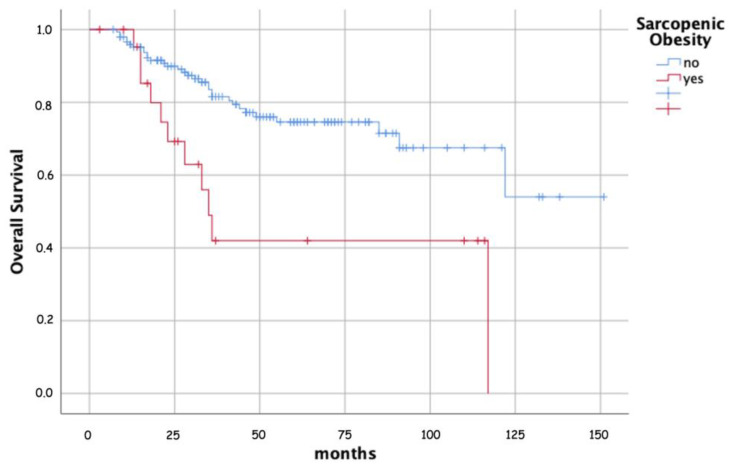
Actuarial survival in patients with and without sarcopenic obesity.

At multivariable analysis, advanced age demonstrated a significant correlation with decreased DMFS and OS rates (*p* = 0.007 and *p* < 0.001, respectively). Furthermore, when comparing Hb values, those exceeding 12 g/dL, as opposed to patients with Hb levels below 10 g/dL, exhibited notably enhanced outcomes. This superiority extended to improved LC (*p* < 0.001), DFS (*p* = 0.007), and OS (*p* = 0.003) rates. Comparatively, patients with FIGO stage III and IV LACC demonstrated inferior outcomes in contrast to those with FIGO stage I–II tumors. The negative correlations for stage III were maintained with DMFS (*p* = 0.006), DFS (*p* = 0.011), and OS (*p* = 0.002). Also, the adverse correlation between nodal metastases and DMFS in univariate analysis was not confirmed in multivariate analysis, while the independent correlation between higher cumulative doses of concurrent CRT plus BRT with improved OS rates was confirmed (*p* = 0.016). Furthermore, in multivariate analysis, both BMI and SP did not show statistically significant correlations with any of the endpoints analyzed. However, the condition of SO was found to be related to substantially worse DFS (*p* = 0.020) and OS (*p* = 0.009) (Table 2, Figure 2).

## 4. Discussion

The purpose of this study was to investigate the prognostic significance of various nutrition-related parameters in LACC patients treated with CRT. Our study confirmed the prognostic impact of several well-established factors in LACC. In fact, advanced age has been consistently associated with poorer outcomes in LACC patients, and our findings align with this evidence [30]. Similarly, lower Hb levels have been recognized as a negative prognostic factor in LACC patients [7,8,9,10], and our analysis corroborated these findings. Furthermore, the FIGO stage has long been known to be a crucial determinant of survival [5,6], and our results confirmed the significant impact of FIGO stage on various clinical endpoints. Finally, our analysis showed that higher RT doses were correlated with improved overall survival, consistent with the principle that an adequate radiation dose is essential for effective tumor control [31].

In this study, we extended the investigation to include body composition-related parameters such as BMI, SP, and SO. These parameters have gained attention in recent years as potential prognostic factors in cancer treatment, including cervical cancer [11,12]. Previous studies have explored the impact of SP on outcomes in LACC patients undergoing CRT. The findings from our study differ from some existing evidence, as we observed that SP did not show a statistically significant correlation with survival outcomes. In fact, the studies by Kiyotoki et al. [13] and Abe et al. [14] identified SP as a significant predictor of DFS and OS, respectively. However, it is important to note that these studies had different definitions and cut-offs for SP, highlighting the need for standardized criteria in future research.

In terms of BMI, our univariate analysis indicated a significant association with DMFS, DFS, and OS, with normal-weight patients showing the highest survival rates. These findings align with studies by Gnade et al. [16] and Clark et al. [17], which also reported worse OS in both underweight and overweight/obese individuals with LACC. However, it is important to note that we observed these associations only in the univariate analysis level. On the contrary, in multivariable analysis, the impact of BMI did not reach statistical significance, indicating that other factors may confound this relationship. The influence of obesity on prognosis in LACC remains a complex and debated topic, as evidenced by the contrasting results reported by Münstedt et al. [18] and Legge et al. [32]. Future studies should continue to explore the relationship between BMI and LACC outcomes, considering potential confounding factors and examining specific subgroups.

Notably, our study is among the first to investigate the significance of SO in LACC patients. We found that patients with SO had significantly worse outcomes in terms of DFS and OS compared to those without this condition. This underscores the importance of evaluating both muscle mass and body fat in cancer prognostication, as SO represents a distinct phenotype with unique clinical implications [11].

The differences observed in the impact of SP, BMI, and SO on LACC outcomes may be attributed to the complex interplay of these parameters with the tumor microenvironment, treatment response, and patient characteristics. While SP and BMI provide valuable information about body composition, SO takes into account the combined effect of muscle loss and excess body fat, potentially reflecting a more comprehensive assessment of a patient’s status and health. In particular, the potential mechanisms underlying the adverse effects of SO on survival may include altered drug pharmacokinetics [28,33], increased systemic inflammation [34,35,36], and impaired immune responses [37,38]. However, further research is needed to elucidate these mechanisms and better understand the clinical implications of SO in cervical cancer.

Our study has some limitations, including its retrospective nature, which may introduce biases. Additionally, the relatively limited number of patients, especially in certain sub-analyses, could affect the generalizability of our findings. Moreover, while our study provides valuable insights into the relationship between sarcopenic obesity and cervical carcinoma prognosis through the assessment of muscle mass, it does not incorporate muscle strength evaluation. The exclusion of muscle strength, a critical component of sarcopenia as highlighted by the Cruz Jantoft algorithm [39], may limit the comprehensiveness of our sarcopenia assessment. Recognizing this limitation, future research should aim to include a holistic evaluation of sarcopenia that encompasses both muscle mass and muscle strength to fully understand its impact on patient outcomes. Nevertheless, a notable strength of our study is the comprehensive evaluation of multiple clinical, tumor-related, and body composition parameters. Moreover, the multidisciplinary approach allowed for a thorough analysis of relevant factors.

Considering the conflicting results of studies on the relationship between BMI and LACC outcomes, further analyses on this topic, considering potential confounding factors and examining specific subgroups, are warranted. Moreover, future research in this area could also investigate the possibility of treating SO in LACC patients, recognizing the challenges posed by the short time frame for planning and delivering CRT. Evaluating whether an improvement in this parameter could translate into improved treatment outcomes is also of particular interest. Furthermore, understanding the dynamics of SO and its response to interventions within the context of LACC treatment could inform the development of predictive models. These models could incorporate SO along with other potential prognostic factors, such as indices of systemic inflammation [22,23], results from functional imaging [40], radiomic indices [41,42,43], and results of liquid biopsies [44,45]. By comprehensively assessing how these factors interact and influence treatment outcomes, we may move closer to achieving more accurate prognostication and personalized treatment strategies for LACC patients. Finally, recognizing the intricate interplay between sarcopenic obesity and treatment outcomes, further studies are warranted to assess changes in SO over the course of treatment. Such research would illuminate potential bidirectional relationships between body composition and treatment efficacy, side-effects, and overall prognosis, contributing significantly to personalized patient care in LACC.

## 5. Conclusions

Our study confirmed the prognostic significance of well-established factors in LACC, including age, Hb levels, FIGO stage, and RT dose. Additionally, we provided insights into the impact of nutrition-related parameters, such as BMI, SP, and SO, on treatment outcomes. SO, in particular, emerged as a novel and significant predictor of adverse outcomes in LACC patients. These findings underscore the importance of considering both muscle mass and body fat in cancer prognostication and treatment planning. Further research is warranted to validate these results and explore potential mechanisms underlying the observed associations. Ultimately, such investigations may contribute to the development of more personalized and effective therapeutic approaches for LACC patients.

## Figures and Tables

**Table 1:** Patients’ characteristics and univariate analysis; survival outcomes are presented in percentage format.

Variable	Value	Patients No	2 y LC	5 y LC	*p*	2 y DMFS	5 y DMFS	*p*	2 y DFS	5 y DFS	*p*	2 y OS	5 y OS	*p*
Age (years)	<55	77	84.2	82.3	0.909	84.7	83.1	**0.049**	70.3	66.7	0.418	90.2	79.3	**0.003**
	55 ≤ age < 70	62	81.1	81.1		82.2	72.6		73.0	66.1		87.7	69.7	
	≥70	34	83.7	83.7		64.8	60.5		59.5	55.2		79.2	49.4	
cN stage	0	102	86.9	85.3	0.271	86.1	81.6	**0.045**	75.0	68.9	0.101	87.2	75.1	0.194
	1–2	71	77.3	77.3		70.8	64.7		61.0	57.1		87.5	64.6	
Total dose (Gy)	≤75	129	81.1	81.1	0.317	79.1	73.4	0.899	67.7	62.0	0.596	83.9	66.6	**0.012**
	>75	44	88.4	85.8		81.8	77.0		72.7	68.0		97.4	81.6	
FIGO stage	I–II	77	93.4	91.5	**0.005**	90.3	85.1	**0.021**	82.2	74.8	**0.003**	93.3	80.5	**0.032**
	III	73	77.7	77.7		71.1	63.8		61.2	56.6		84.4	58.8	
	IV	23	64.2	64.2		72.8	72.8		49.7	49.7		74.7	68.5	
Maximum tumor diameter	≤4	55	91.9	89.0	0.114	84,3	75.9	0.910	77.0	68.4	0.403	88.3	74,3	0.675
	>4	118	78.8	78.8		77.8	74.2		65.5	62.0		87.0	69.1	
Histological type	SCC	139	82.8	81.6	0.598	80.0	74.4	0.799	69.1	63.6	0.917	89.0	72.3	0.305
	N-SCC	34	84.4	84.4		80.0	76.6		69.6	66.4		81.6	63.9	
Overall treatment	≤54	92	83.4	81.7	0.888	78.1	73.1	0.536	68.4	63.7	0.892	86.1	75.9	0.254
	>54	81	82.8	82.8		82.0	76.6		70.0	64.5		88.8	65.5	
Hemoglobin	<10	16	49.2	49.2	**<0.001**	72.3	72.3	0.270	48.1	48.1	**0.007**	55.0	55.0	**0.040**
	10 ≤ Hb < 12	42	73.3	69.7		79.5	79.5		63.4	60.1		79.5	66.8	
	≥12	115	91.4	91.4		81.4	73.9		74.3	67.7		94.2	73.9	
Body Mass Index	<18.5	7	85.7	85.7	0.247	71.4	71.4	**0.033**	71.4	71.4	**0.018**	85.7	42.9	**0.023**
	18.5 ≤ BMI < 25	90	89.9	88.3		87.0	85.6		79.0	75.8		91.9	79.9	
	25 ≤ BMI < 30	49	74.4	74.4		72.8	62.7		60.5	52.1		82.1	61.3	
	≥30	27	75.5	75.5		71.1	60.2		51.9	46.7		82.5	56.2	
Sarcopenia	≤7.53	19	94.7	94.7	0.175	77.5	77.5	0.902	77.5	77.5	0.284	94.7	78.9	0.266
	>7.53	153	81.5	80.5		80.1	74.2		67.9	62.3		86.4	69.4	
Sarcopenic obesity	0	148	85.4	84.3	**0.066**	81.8	77.7	0.110	73.3	68.3	**0.005**	89.9	74.6	**<0.001**
	1	23	66.7	66.7		69.9	55.9		43.0	36.8		69.2	42.0	

Legend: BMI: body mass index; DFS: disease-free survival; DMFS: distant metastasis-free survival; FIGO: International Federation of Gynecology and Obstetrics; HR: hazard ratio; LC: local control; OS: overall survival; y year.

**Table 2:** Multivariable analysis; only statistically significant values are shown.

Parameter	Values	Patients N (%)	LC			DMFS			DFS			OS		
			HR	_95%_CI	*p*	HR	_95%_CI	*p*	HR	_95%_CI	*p*	HR	_95%_CI	*p*
Age (years)	<55	77				1	rif.	**0.024**				1	rif.	**0.003**
	55 ≤ age < 70	62				1.336	0.642–2.781	0.438				2.052	0.936–4.502	0.073
	≥70	34				2.919	1.334–6.388	**0.007**				4.403	1.878–10.322	**<0.001**
Total dose (Gy)	≤75	129										1	rif.	**0.016**
	>75	44										0.368	0.163–0.831	
FIGO stage	I–II	77	1	rif.	0.083	1	rif.	**0.019**	1	rif.	**0.031**	1	rif.	**0.008**
	III	73	2.128	0.858–5.227	0.103	2.664	1.318–5.384	**0.006**	2.148	1.187–3.884	**0.011**	3.077	1.515–6.250	**0.002**
	IV	23	3.210	1.135–9.083	**0.028**	2.676	0.974–7.352	**0.056**	2.124	0.959–4.707	0.063	2.496	0.873–7.139	0.088
Hemoglobin	<10	16	1	rif.	**<0.001**				1	rif.	**0.024**	1	rif.	**0.010**
	10 ≤ Hb < 12	42	0.395	0.155–1.009	0.052				0.394	0.168–0.922	0.032	0.400	0.147–1.087	0.072
	≥12	115	0.129	0.049–0.340	**<0.001**				0.347	0.161–0.747	**0.007**	0.227	0.086–0.601	**0.003**
Sarcopenic obesity	0	148	1	rif.	0.071				1	rif.	**0.020**	1	rif.	**0.009**
	1	23	2.257	0.933–5.457					5.289	1.298–21.546		2.645	1.275–5.488	

Legend: **_95%_**CI: 95% Confidence Interval; BMI: body mass index; DFS: disease-free survival; DMFS: distant metastasis-free survival; FIGO: International Federation of Gynecology and Obstetrics; HR: hazard ratio; LC: local control; OS: overall survival.

## Data Availability

Data supporting the reported results will be made available upon reasonable request.
